# Ultrasound Biomicroscopy and Scheimpflug Imaging in Anterior Megalophthalmos: Changes Seen after Cataract Surgery

**DOI:** 10.1155/2015/195950

**Published:** 2015-05-07

**Authors:** Nishant Nawani, Arun K. Jain, Ramandeep Singh

**Affiliations:** Department of Ophthalmology, Advanced Eye Centre, Postgraduate Institute of Medical Education and Research, Chandigarh, India

## Abstract

*Purpose*. With this report we describe ultrasound biomicroscopic (UBM) findings in a patient with anterior megalophthalmos before and after undergoing phacoemulsification with posterior chamber intraocular lens implantation. *Methods*. Phacoemulsification was carried out for nuclear sclerosis in both eyes of a patient diagnosed with anterior megalophthalmos. The patient was subjected to detailed ophthalmic examination including ultrasound biomicroscopy and Scheimpflug imaging prior to and after surgery. Preoperative ultrasound biomicroscopy revealed a deep anterior chamber with posterior bowing of the midperipheral iris in both eyes. The ciliary processes were inserted on the posterior surface of the iris. UBM was repeated postoperatively as well. *Results*. Phacoemulsification and posterior chamber intraocular lens implantation (IOL) were carried out successfully in both eyes. The IOLs were well centered and captured within the anterior capsulorhexis. The anterior chambers were hyperdeep, 6.24 mm (OD) and 6.08 mm (OS), respectively. The posterior bowing of the midperipheral iris was absent, with the iris having a more flat profile. *Conclusion*. UBM findings in anterior megalophthalmos seemed to partially resolve after cataract surgery. The anterior chamber deepens appreciably as well.

## 1. Introduction

Anterior megalophthalmos is a rare, mostly X-linked recessive condition with findings of a horizontal corneal diameter greater than 13.0 mm, ciliary ring enlargement, anterior embryotoxon, mosaic corneal dystrophy, Krukenberg's spindle, hyperdeep anterior chamber, iris hypoplasia, large capsular bag, cataract, and lens subluxation [1, 2]. Cataract surgery in anterior megalophthalmos is challenging because of a deep anterior chamber, enlarged ciliary ring, weakened zonules, and large capsular bag. Preoperative UBM scanning is an important tool to assess the zonules. A previous report describes ciliary body dysplasia with thinning of the root of the iris and insertion of ciliary processes on the posterior surface of the peripheral iris [3]. We documented similar features and described the changes seen after cataract surgery. To the best of our knowledge, changes in UBM features have not been described after cataract surgery in anterior megalophthalmos.

## 2. Case Report

**Figure 1:**
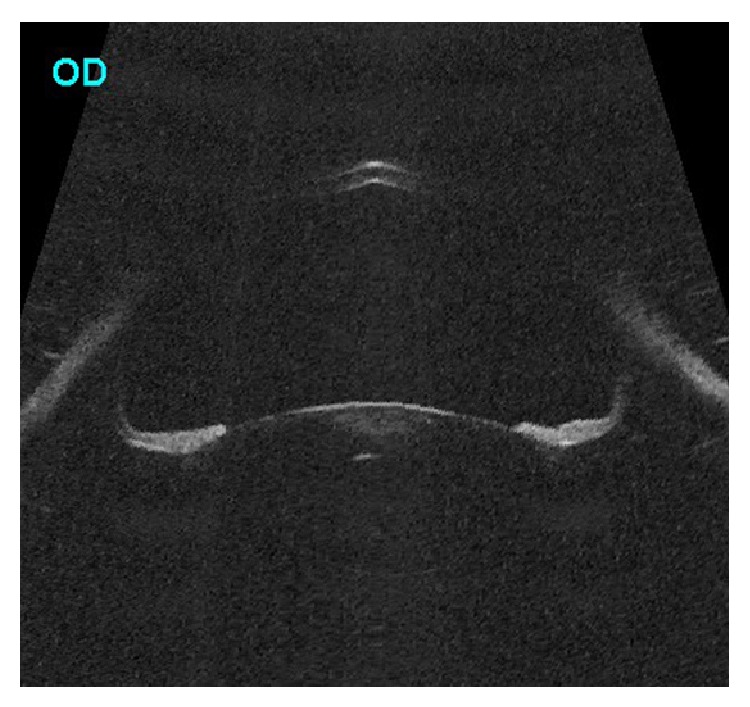
Ultrasound biomicroscopy (axial scan) of the right eye showing hyperdeep anterior chamber with prominent posterior bowing of midperipheral iris with crystalline lens touching the iris.

**Figure 2:**
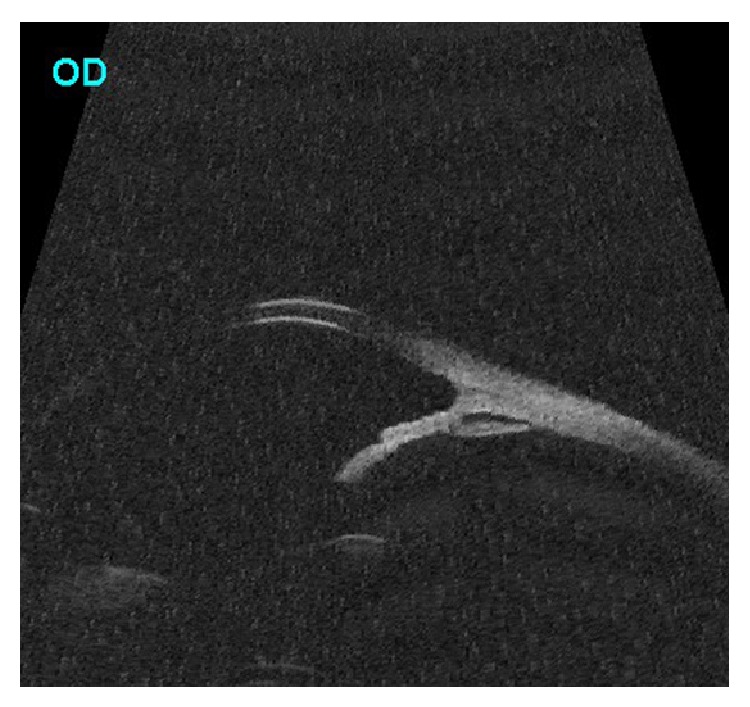
Radial section of ultrasound biomicroscopy showing insertion of ciliary processes on the posterior surface of iris and posterior bowing of iris.

**Figure 3:**
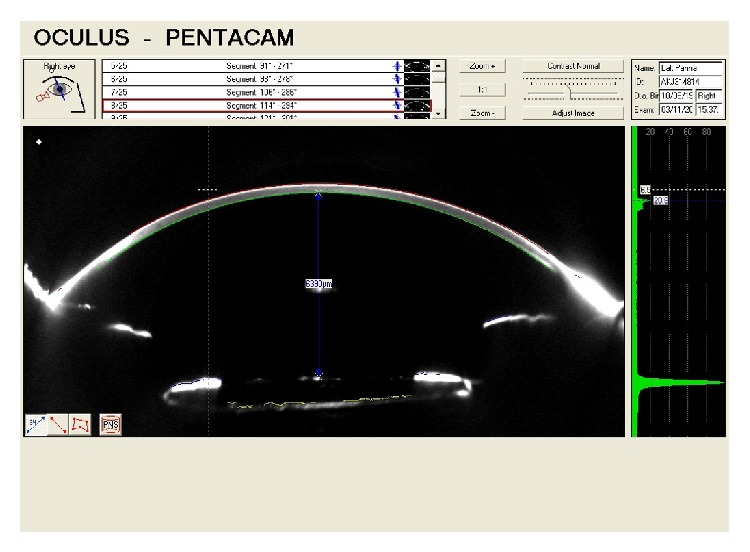
Scheimpflug image of the right eye after cataract surgery shows hyperdeep anterior chamber.

**Figure 4:**
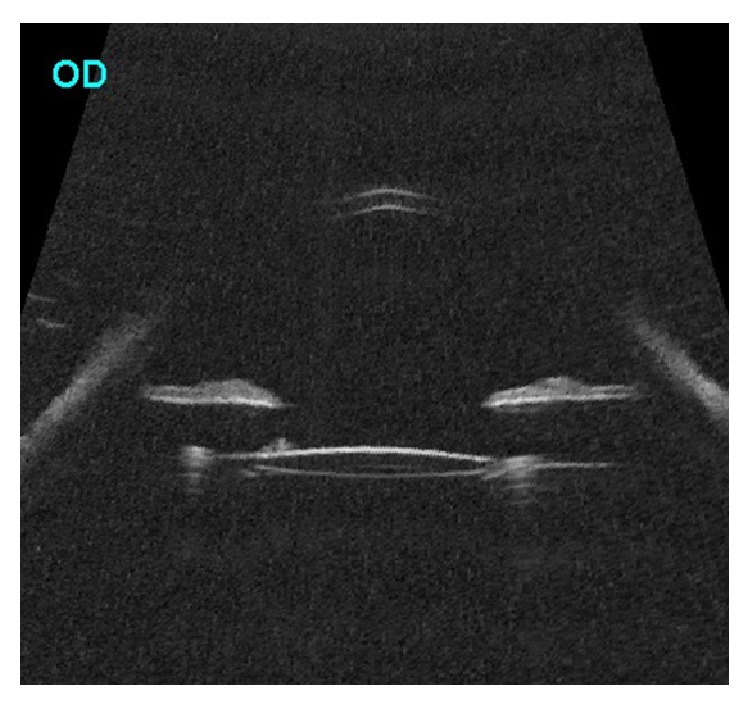
Ultrasound biomicroscopy (axial scan) of the right eye after cataract surgery showing resolution of posterior bowing of midperipheral iris and well-centered intraocular lens with gap between lens and iris.

A 42-year-old man reported to us with chief complaints of painless, decreased vision in both eyes of 4-month duration. His best corrected visual acuity (BCVA) was 6/36 (OD) and 6/36 (OS) with myopic correction in both eyes. There were nuclear sclerosis in both eyes associated with iridodonesis and phacodonesis and deep anterior chambers. There was prominent posterior bowing of the iris in the midperiphery associated with stromal atrophy. Gonioscopy revealed dense pigmentation on the trabecular meshwork. Optic disc and retinal examination was normal in both eyes. The intraocular pressure (IOP) by Goldmann applanation was 16 mmHg in the right eye and 14 mmHg in the left eye. Pentacam (Oculus) revealed anterior chamber depth to be 5.77 mm (OD) and 5.54 mm (OS) with central corneal thickness of 478 *μ*m and 498 *μ*m, respectively. Ultrasound biomicroscopy revealed prominent posterior bowing of the midperipheral iris, scanty zonular support, and ciliary processes inserted on the posterior surface of iris (Figures 1 and 2) in both eyes. These findings were consistent for 360° of the ciliary ring, similar to a previous case report [3]. The axial length was 25.22 mm (OD) and 24.40 mm (OS) and white-to-white corneal diameters were 13.2 mm and 13.1 mm, respectively. A-scan revealed lens thickness of 4.71 (OD) and 4.72 mm (OS). Vitreous cavity measured 14.74 mm and 14.14 mm, respectively. Normal vitreous index is about 69% [4]. This patient had vitreous index of 58% in both eyes. The postlimbal anterior chamber depth is 0.20 mm in a 20-year-old, which reduces to zero by the age of 50 years [4]. In our patient, the postlimbal depth was 1.9 mm (OD) and 1.8 mm (OS), indicating an enlarged anterior segment in both eyes. A diagnosis of anterior megalophthalmos was made and phacoemulsification with posterior chamber intraocular lens implantation (PCIOL) was performed first in the right eye followed by the left four weeks later. The IOL power calculations and procedure of phacoemulsification were reported by the authors in a previous report [5]. SRK II formula was used for biometry and 2 dioptres (D) was added to the emmetropic IOL power to err towards myopic postoperative refraction [5]. Phacoemulsification was performed through scleral tunnel in both eyes. A three-piece, acrylic hydrophobic IOL was implanted into the sulcus in both eyes, with rhexis optic capture technique [6]. In both eyes, the incision was left sutureless and there was no wound leak postoperatively. There were no surgical complications and at all follow-up visits the IOLs were well centered in both eyes. Postoperatively, Scheimpflug imaging showed deeper anterior chamber in both eyes, 6.38 mm in the right eye (Figure 3) and 6.08 mm in the left eye. UBM (Figure 4) revealed a well-centered IOL without the posterior bowing of the peripheral iris. The iris profile was flat the and there was a significant distance between the iris and the anterior surface of the IOL, due to an increased postlimbal anterior chamber depth, as found in anterior megalophthalmos. The ciliary processes, however, were still inserted on the posterior surface of the iris for 360°.

## 3. Discussion

Cataract surgery and IOL implantation in anterior megalophthalmos are challenging. The authors have previously reviewed literature on phacoemulsification in anterior megalophthalmos and have described a novel technique for IOL centration in such cases [5]. They had found that capturing the IOL optic through a round and centered anterior capsulorhexis results in good centration of IOL optic. Vaz and Osher [7] implanted custom IOLs with a diameters of 16 mm in both eyes of an anterior megalophthalmos patient whose corneal dimensions were 16.25 mm in the right eye and 16.50 mm in the left eye. The IOLs remained well centered postoperatively. Since it was not possible for authors to obtain customized IOLs, they described the rhexis capture technique for IOL centration [5].

The refractive outcomes of phacoemulsification in anterior megalophthalmos in recent reports have shown a postoperative hyperopic refractive surprise [7, 8]. Vaz and Osher reported off-target hyperopic postoperative refraction of 2.9 and 2.25 dioptres (D) in both eyes of their patient [7]. Assia et al. [8] aimed for a myopic (−0.65 D) refraction for the right eye of their patient and achieved a final refraction of +2.25 D spherical equivalent. They then targeted −1.25 D for the left eye of the same patient and achieved a postoperative refraction of +1 D. After reviewing these reports we added 2 D to the emmetropic IOL power as calculated by SRK II formula. Postoperatively we achieved planorefraction (OD) and −0.75 D cylinder ×90°(OS).

This is the first report which describes the changes in UBM features following cataract surgery in such patients. UBM revealed that the ciliary processes were still inserted on the posterior surface of the iris postoperatively. However, the preoperative feature of prominent posterior bowing of the midperipheral iris was absent. The iris configuration was much flattened out. We hypothesize an explanation for this finding. In a normal 40-year-old individual the crystalline lens would weigh about 192 mg [9]. The product catalogue of the acrylic hydrophobic IOL, Ar40e (Sensar Optiedge, AMO), implanted in this patient described the weight of the IOL in air to be 23.1 mg. UBM is performed in a supine, gravity dependent position. A heavier crystalline lens would exert more pull on the zonule-ciliary body complex, part of which is already inserted on the posterior surface of the iris (in our patient), than a much lighter IOL, hence the absence of the posterior bowing of the peripheral iris postoperatively. Another incident postoperative finding, both clinically and on UBM, is the relatively large distance between the iris and the IOL. This is attributable to an enlarged ciliary ring and greater postlimbal anterior chamber depth.

In conclusion, preoperative UBM is helpful in assessing the zonular status in patients of anterior megalophthalmos with cataract before they undergo cataract surgery and postoperatively reveals any changes in anatomy of the iris/ciliary body. Pentacam imaging provides additional information about the corneal thickness and anterior chamber depth, which is helpful while planning cataract extraction in patients with anterior megalophthalmos.
